# microRNA based prognostic biomarkers in pancreatic Cancer

**DOI:** 10.1186/s40364-018-0131-1

**Published:** 2018-05-21

**Authors:** Shixiang Guo, Andrew Fesler, Huaizhi Wang, Jingfang Ju

**Affiliations:** 1 Stony Brook University, Stony Brook, New York, 11794 USA; 2 Third Military Medical University, Chongqing, 400038 People’s Republic of China

**Keywords:** Pancreatic ductal adenocarcinoma, miRNA, Biomarkers, Resistance, Prognosis, Chemotherapy, Gemcitabine

## Abstract

Despite tremendous research efforts focused on diagnosis and treatment, pancreatic ductal adenocarcinoma remains the third leading cause of cancer-related death in the United States, with a 5-year overall survival rate of less than 5%. Although resistance is rather complex, emerging evidence has demonstrated that epigenetic alterations (e.g. miRNA) have important roles in PDAC progression as well as resistance to therapy. Certain miRNAs have been identified as potential prognostic biomarkers in PDAC. In this review, we summarize the recent developments in miRNA research related to PDAC therapeutic resistance mechanisms and the potential of miRNAs as prognostic biomarkers for future clinical management of PDAC.

## Background

Pancreatic ductal adenocarcinoma (PDAC) is the third deadliest cancer in the United States [1]. It is characterized by late clinical presentation, early metastasis and poor prognosis [2]. A large proportion of patients are diagnosed with locally advanced or metastatic disease at the time of presentation [3]. Current therapy for PDAC mainly involves surgical resection, adjuvant chemotherapy and radiotherapy [4]. Despite the advancement in clinical management (e.g. Abraxane), patient outcomes remain unsatisfactory [5, 6].

In addition to patients presenting with advanced disease, many patients also experience early appearance of post-operative recurrence [7]. Therefore, adjuvant treatments (chemotherapy, radiotherapy etc.) are necessary and critical for management of patients with advanced disease. However, few effective chemotherapeutic options exist for advanced PDAC patients in the clinic. Since 1997, gemcitabine has been approved as the standard first-line chemotherapeutic, several novel therapeutic regimens based on gemcitabine have also been investigated for PDAC treatment [8]. Multiple agents have been assessed in combination with gemcitabine including 5-fluorouracil, oxaliplatin, cisplatin and capecitabine [9–12]. However, the impact on patient survival is rather limited. Such failure is caused, at least in part, by chemoresistance. Chemoresistance is mainly classified into intrinsic and acquired resistance. Compared with intrinsic resistance where therapy is ineffective from the start of treatment, acquired resistance with continuous chemotherapy ultimately causes relapse and metastasis [13]. Over the past decade extensive research efforts have been dedicated to investigate the underlying mechanisms of chemoresistance. Resistance involves PDAC stem cells which have unique characteristics including enhanced epithelial–mesenchymal transition (EMT), autophagy, and altered metabolism that contributes to their plastic nature and chemoresistant phenotype. Altered expression of many different genes (e.g. KRAS, TP53, CCND1, BCL-2, BIRC5) and changes in key signaling pathways (e.g. Notch, PI3K/AKT, NF-κB, Hedgehog, cell cycle, apoptosis) also contribute to resistance [13–16]. Clearly there is an urgent need to develop early detection and/or novel prognostic biomarkers to help better manage PDAC treatment to maximize survival benefits and to avoid toxicity.

## Epigenetic regulations mediated by miRNAs in PDAC resistance mechanism

Based on a large body of growing evidence, we know PDAC resistance is regulated, at least in part, by epigenetic alterations including miRNA. miRNAs are small non-coding RNAs 18–22 nucleotides in length that have been identified to be associated with tumorigenesis, cell cycle control, apoptosis, proliferation, chemoresistance, invasion and metastasis [17]. In PDAC, miRNAs have been demonstrated to modulate key targets and pathways such as KRAS, TP53, PI3K/AKT, NF-κB and Hedgehog signaling, and their aberrant expression is associated with chemoresistance (14). It has been shown that miR-17-92 cluster counteracts quiescence and chemoresistance in a distinct subpopulation of pancreatic cancer stem cells by acting through the NODAL/ACTIVIN/TGF-β1 signaling cascade [18]. A number of important miRNAs in PDAC are listed in Table 1.

**Table 1:** Critical miRNAs as potential diagnostic, therapeutic, prognostic targets in PDAC

miRNAs	Function	Expression in tumor	Targets	Pathway	Ref.
miR-21	Oncogenic	Up-regulated	PTEN, PDCD4, CDK6, CDKN1A, IL-6R, FAS, TPM1, APAF1, SOCS5	PI3K/AKT	[20–22]
miR-34	Tumor suppressor	Down-regulated	NOTCH, BCL2, VEGFA, CCND1, CDK6	p53/p38-MAPK/NOTCH PI3K/AKT	[14, 24, 26]
miR-200 family	Tumor suppressor	Down-regulated	E-cadherin, ZEB, Vimentin	NOTCH, EMT	[14, 20, 23]
Let-7 family	Tumor suppressor	Down-regulated	KRAS, HRAS, LIN28, HMGA2, NF2, TRIM71	EMT, KRAS	[16]
miR-15a	Tumor suppressor	Down-regulated	WANT3A, FGF7, BMI-1	ERK/AKT, EMT	[28, 29]
miR-506	Tumor suppressor	Down-regulated	SPHK1, PI3M	SPHK1/AKT/NF-κB	[31, 32]
miR-221	Oncogenic	Up-regulated	KIT, CDKN1C, CDKN1B	EMT, PKC/NF-κB, PTEN/PI3K/AKT	[14, 21]
miR-96	Tumor suppressor	Down-regulated	KRAS, AKT	KRAS, PI3K/AKT	[14, 15]
miR-17-92	Tumor suppressor	Down-regulated	p21, p57, TBX3	NODAL/ACTIVIN/TGF-1	[18]
miR-145	Tumor suppressor	Down-regulated	KRAS, RREB1	KRAS, PI3K/AKT	[14]
miR-155	Oncogenic	Up-Regulated	TP53INP	Apoptosis, Exosome Synthesis	[30]

In terms of resistance of PDAC to chemotherapeutic treatment, miR-21 is one of the most investigated oncogenic miRNAs related to gemcitabine resistance. Elevated expression of miR-21 inhibits the anti-tumor activity of gemcitabine, and is significantly associated with shorter survival time [19]. Giovannetti et al. suggests that miR-21 contributes to gemcitabine chemoresistance by inhibiting tumor suppressor gene phosphatase and tensin homologue (PTEN), thereby activating the PI3K/AKT pathway [20]. Park et al. illustrated that silencing miR-21 leads to cell cycle arrest (G1 phase) and induction of apoptosis by up-regulating PTEN [21]. Hwang et al. showed that down-regulation of miR-21 expression correlates with prolong overall survival and benefit from chemotherapeutic treatment [22]. In addition to miR-21, several other miRNAs (miR-34, miR-217, miR-96, miR-145) have been shown to be deregulated and impact the PI3K/AKT pathway in PDAC [14]. EMT/ mesenchymal-epithelial transition (MET) has been shown to be critical in chemoresistance of PDAC and is mediated by key miRNAs. Emerging evidence confirms that the miR-200 family plays a key role in chemoresistance via reversing EMT. Ali et al. reported that down-regulation of miR-21 and restoration of miR-200b and miR-200c inactivates pAKT by reactivation of PTEN and reverses EMT, resulted in enhanced gemcitabine sensitivity [19]. Furthermore, Li et al. show that miR-200b, miR-200c, let-7 family (let-7b, let-7c, let-7d, let-7e) are down-regulated in gemcitabine-resistant PDAC cells. Restoration of miR-200 and let-7 results in a reversal of PDAC from EMT to MET and sensitivity to gemcitabine treatment [23].

Previous studies have demonstrated that the miR-34 family (miR-34a, b and c) is associated with p53 and p38-MAPK pathways in response to DNA damage [24]. Down-regulation of miR-34 is responsible for progression of various malignancies including PDAC, lung, breast, prostate and liver cancer [25]. miR-34 has an anti-cancer role via modulating targets implicated in apoptosis, cell cycle, and DNA repair, such as NOTCH, BCL2, VEGFA, CCND1 and CDK6 [26]. In regards to PDAC resistance, Ji et al. reported that miR-34 is regulated by p53, and inhibits target genes NOTCH and BCL-2. Loss of miR-34 leads to the enrichment of cancer stem cells or tumor-initiating cells and restoration of miR-34 inhibits PDAC cell growth and enhanced chemotherapeutic sensitivity to gemcitabine [27].

Zhang et al. suggested that miR-214 enhances chemoresistance to gemcitabine by down-regulating the tumor suppressor gene ING4, while miR-15a can suppress the growth of chemoresistant PDAC cells via targeting WNT3A and FGF7, contributing to progression and proliferation through the phosphorylation of the kinases ERK and AKT [28]. Moreover, Guo et al. indicated that miR-15a inhibits cell proliferation and EMT by down-regulating BMI-1 in PDAC [29].

**Fig. 1:**
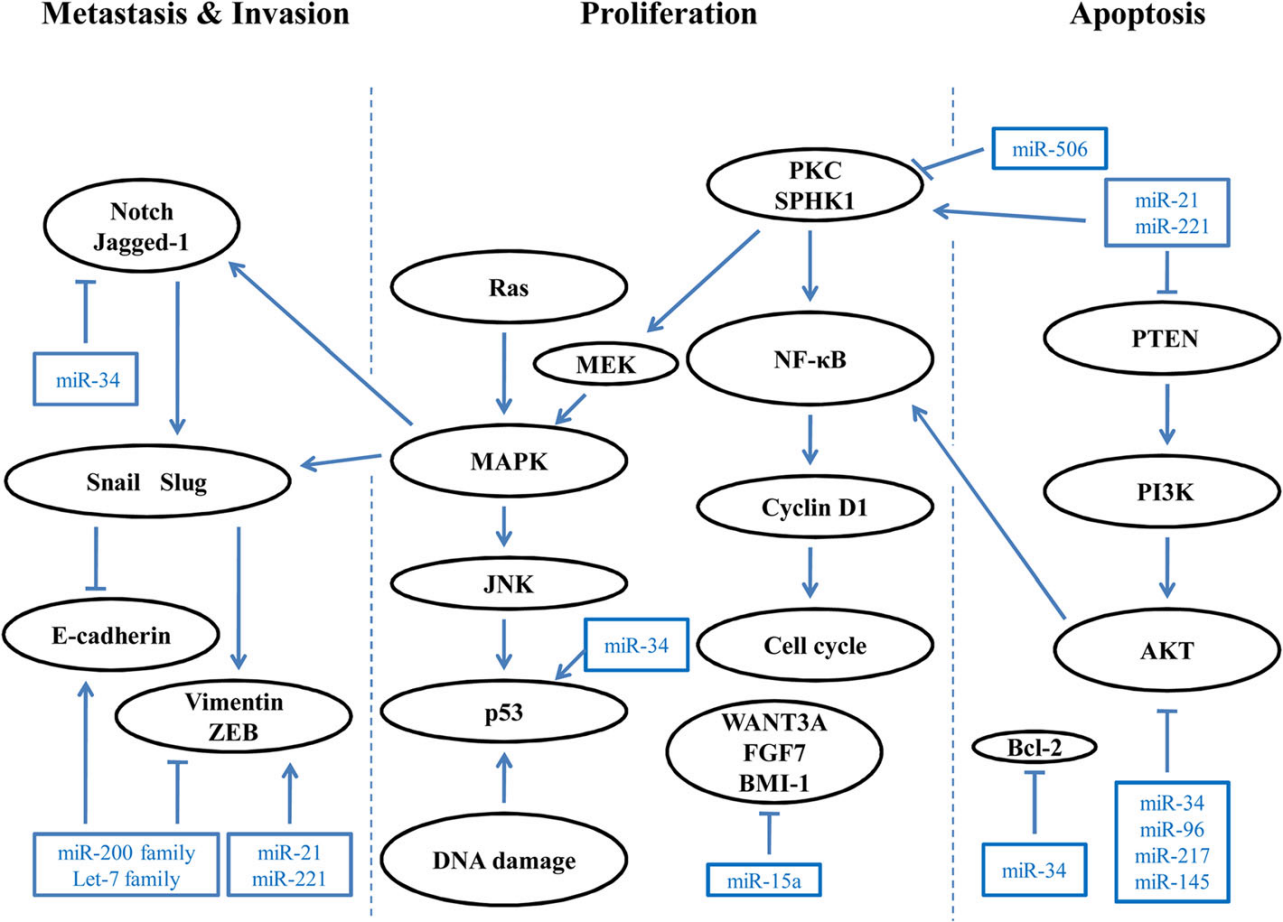
Schematic illustration of miRNAs that are important in PDAC through regulation key targets and signaling pathways

miR-155 expression has been shown to induce gemcitabine resistance. Prolonged exposure to gemcitabine leads to increased miR-155 expression, which inhibits apoptosis and increases exosome production, resulting in gemcitabine resistance [30]. Li et al. reported that miR-506 can inhibit cell proliferation, induce cell cycle arrest, promote apoptosis and enhance chemosensitivity to gemcitabine in PDAC by regulating the SPHK1/AKT/NF-κB signaling pathway [31]. Meanwhile, Du et al. revealed that miR-506 represses PDAC cell proliferation by targeting PIM3, a member of oncogenic PIM family [32]. Based on these studies, it appears that miR-506 plays a tumor suppressor role in PDAC. The functions of several miRNAs in PDAC are shown in Fig. 1.

## miRNAs as prognostic biomarkers

Based on the poor prognosis of PDAC, the development of early detection methods, more effective treatment options and better prognostic biomarkers are of critical significance. Besides the significance of miRNAs for early detection and diagnosis, accumulating evidence suggests that miRNAs have great potential as prognostic biomarkers [33, 34]. Dillhoff et al. showed that 79% of PDAC patients with miR-21 high expression have poor outcomes [35]. Bloomston et al. found that six miRNAs (miR-30a-3p, miR-105, miR-127, miR-187, miR-452, and miR-518a-2) are predictive of better prognosis (survival time beyond 2 years) in PDAC patients [36]. One recent study found that over-expression of miR-212 and miR-675 and down-regulation of miR-148a, miR-187, and let-7 g were independent predictors of worse prognosis in PDAC patients [37]. miR-142-5p and miR-204 are found to be down-regulated in chemoresistant PDAC cells, and high expression of these miRNAS in PDAC patients associates with better overall survival [38]. One study has concluded that miR-155, miR-203, miR-210, miR-222, miR-200c and miR-302 are associated with PDAC patients’ outcome [39]. In our previous studies, we found that low expression of miR-506 was an independent predictor of poor prognosis in PDAC, while miR-15a is significantly related with prognosis of PDAC patients [29, 31]. Collectively, these studies support the potential role of miRNAs as prognostic biomarkers for PDAC.

## Other class of noncoding RNAs in PDAC resistance and prognosis

Beyond miRNA, other types of noncoding RNAs (e.g. lncRNA, circRNA) have also been implicated in cancer resistance and prognosis [40–42]. It has been reported that elevated HOTAIR expression is significantly associated with poor prognosis of PDAC patients. HOTAIR has oncogenic activity by suppressing a number of interferon-related genes and genes related to cell cycle control [43]. Huang et al. recently reported that circular RNA, hsa_circ_0000977, is upregulated in PDAC. Inhibition of hsa_circ_0000977 suppresses PDAC cell proliferation and induces cell cycle arrest. Hsa_circ_0000977 interferes with hsa-miR-874-3p and increases Polo like kinase 1 (PLK1) expression [44]. It is conceivable that we are still at the early stage of exploring other types of noncoding RNAs in PDAC and there will be more exciting discoveries in the future.

## Conclusions

It is clear that PDAC utilizes a variety of mechanisms to maintain a highly resistant phenotype. The highly plastic nature of PDAC resistance is mediated by genetic and epigenetic alterations. The epigenetic controls such as miRNAs allow cells to quickly adapt to the genotoxic stress environment caused by chemotherapy. miRNAs can quickly modulate mRNA translation in PDAC cells in response to chemotherapeutic treatment. As a result, a number of miRNAs have shown great potential as prognostic biomarkers in PDAC. Hopefully these biomarker miRNAs will form a solid foundation to better manage clinical treatment strategies to enhance survival benefits and avoid toxicity. Beyond miRNAs as prognostic biomarkers, as miRNAs are multi-targeted entities that suppress a number of key targets and pathways, some of these miRNAs will be good candidates to develop as novel therapeutics for overcoming PDAC resistance.

## Abbreviations

circRNA: circular RNA
EMT: Epithelial–mesenchymal transition
lncRNA: long noncoding RNA
MET: Mesenchymal–epithelial transition
PDAC: Pancreatic ductal adenocarcinoma
